# Membrane Lipid Remodeling in Response to Salinity

**DOI:** 10.3390/ijms20174264

**Published:** 2019-08-30

**Authors:** Qi Guo, Lei Liu, Bronwyn J. Barkla

**Affiliations:** Southern Cross Plant Science, Southern Cross University, Lismore, New South Wales 2480, Australia

**Keywords:** membrane lipids, lipid regulation, phospholipids, glycolipids, sterols, fatty acids, signaling, lipid metabolism, lipid biosynthesis

## Abstract

Salinity is one of the most decisive environmental factors threatening the productivity of crop plants. Understanding the mechanisms of plant salt tolerance is critical to be able to maintain or improve crop yield under these adverse environmental conditions. Plant membranes act as biological barriers, protecting the contents of cells and organelles from biotic and abiotic stress, including salt stress. Alterations in membrane lipids in response to salinity have been observed in a number of plant species including both halophytes and glycophytes. Changes in membrane lipids can directly affect the properties of membrane proteins and activity of signaling molecules, adjusting the fluidity and permeability of membranes, and activating signal transduction pathways. In this review, we compile evidence on the salt stress responses of the major membrane lipids from different plant tissues, varieties, and species. The role of membrane lipids as signaling molecules in response to salinity is also discussed. Advances in mass spectrometry (MS)-based techniques have largely expanded our knowledge of salt-induced changes in lipids, however only a handful studies have investigated the underlying mechanisms of membrane lipidome regulation. This review provides a comprehensive overview of the recent works that have been carried out on lipid remodeling of plant membranes under salt treatment. Challenges and future perspectives in understanding the mechanisms of salt-induced changes to lipid metabolisms are proposed.

## 1. Introduction

**Figure 1:**
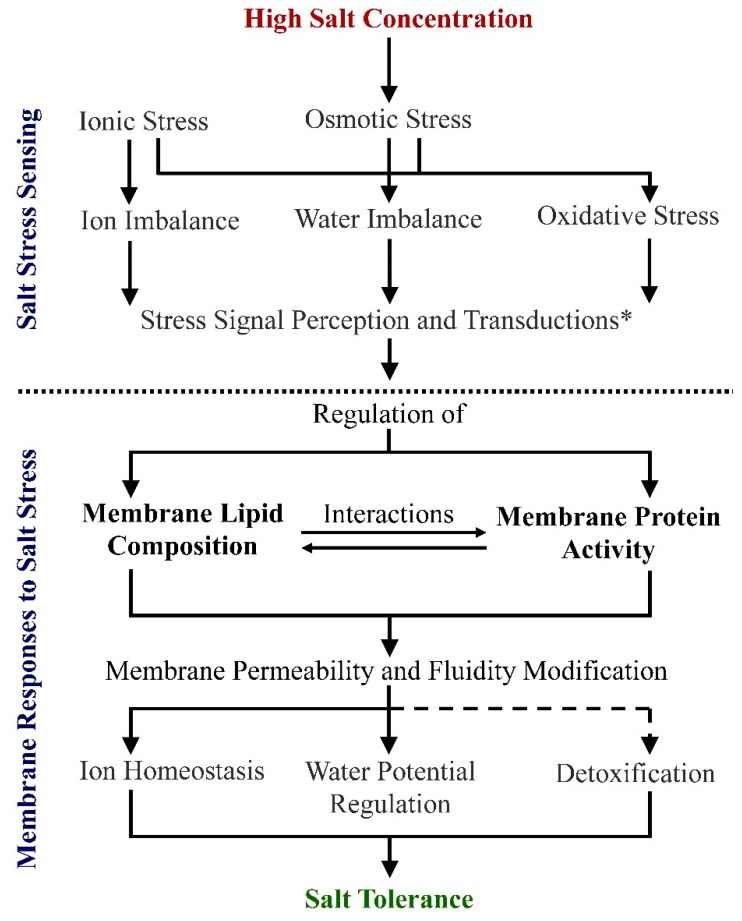
Salt stress mechanisms and membrane responsive regulations of membranes in plant cells. Dotted line separates mechanisms of salt sensing from downstream response mechanisms. Dashed line indicates pathway has not yet been verified. The model presented is based on data from [10,11,15]. * Signaling includes shared signaling pathways and individual signaling pathways induced from different primary stress responses

Soil salinization is a major environmental concern, affecting more than 800 million hectares of land, equivalent to over 6% of the world’s total land surface. It is estimated that approximately 15% of total cultivated lands show some level of degradation due to soil salinization, severely reducing crop production potential [1]. Worse still, human activities, such as vegetation clearance, land irrigation, and fertilizer mismanagement have been accelerating soil salinization [2,3,4,5]. This is exasperated by the fact that the salt tolerance of most crops is poor; in general, staple crops such as wheat, rice, and maize show significant yield reductions in the presence of salt [6,7,8,9]. To overcome this problem, there is a need for screening for more salt-tolerant cultivars, engineering crops to increase salt tolerance, or diversifying our agricultural systems to cultivate more salt-tolerant under-utilized crop plants. These approaches would help to secure crop yields and sustain food production, as well as allow continued use of marginal land. However, for this to happen, an increased understanding of the range of molecular mechanisms used by plants to tolerate salt is necessary. It is well known that salt stress comprises three main elements: Ion imbalance, which can cause nutrient stress; hyperosmotic stress, which affects the uptake and movement of water; and oxidative damage due to the formation of superoxide radicals [10,11,12,13,14] (Figure 1). It is interesting that while cellular membranes are integral to all these components of salt-stress, lipids are rarely mentioned in reviews that discuss mechanisms of salt stress.

Evidence for membrane remodeling has been shown in plants exposed to salinity, with measured changes in permeability recorded [16,17,18]. Changes in membrane properties likely involve the remodeling of membrane lipids (Figure 1), as has been documented for stress-induced changes in the composition of membranes from animal, yeast, bacteria, and plant cells [19,20,21,22].

Studies of plant membrane lipid alterations in the presence of NaCl have mainly focused on changes in total membrane lipids or specifically focused to the plasma membrane (Table 1, Table 2 and Table 3 and references therein). Although evidence has shown that other endomembranes may also be affected by plant salt tolerance [23,24], very little information is available. In addition, while several studies have demonstrated the salt-induced changes to enzymes involved in lipid metabolism at the level of gene expression (Section 4), they do not provide data on how these changes could govern the membrane lipid content in response to salinity. This review summarizes the current knowledge about membrane lipid remodeling of plants in response to salinity stress, with a focus on the gaps, challenges, and future perspectives in understanding salt-responsive mechanisms of plant membrane lipids.

## 2. Plant Lipids

Biological membranes are a mixture of many different types of lipids, and their relative amounts and composition differs between functionally distinct domains and between organelles and vesicles [42]. Generally, a single plant cell would contain over 1000 lipid species [43]. However, despite the advancements in analytical technology, MS methods are still not advanced sufficiently to allow a comprehensive characterization of all lipid species [44,45], and work in this area primarily focuses on the major lipid classes.

Plant membrane lipids belong to three major groups: Sphingolipids, sterols, and glycerolipids [46]. Sphingolipids have a ceramide backbone, which consists of a fatty acid amidated to a long-chain base (LCB) with a polar head attached to the alcohol residue. In plants, sphingolipids are recognized as general components of membranes, influencing membrane integrity and permeability [47,48]. It is estimated that there are at least 500 different molecular species of sphingolipids in plant cells [49]. However, the significance of this structural complexity is unclear [50]. Recent interest in sphingolipids has focused on their role in pollen development and signal transduction in plants under abiotic stress [51]. For instance, chilling stress in *Arabidopsis thaliana* seedlings led to a significant decrease in total LCB lipids, which mainly reflected a decline in membrane sphingolipids since the most abundant free LCB lipids remained unchanged [52]. Meanwhile, overexpression of the non-symbiotic haemoglobin AHb1, a major cold signaling molecule, resulted in an increased level of LCBs, suggesting the regulation of sphingolipids related to cold stress signaling [52,53].

Sterols refers to an isoprenoid that consists of a cyclopenta phenanthrene made up of four rigid rings and hydroxylated at position *sn-3*. Higher plant cells contain a vast array of sterols, in particular 61 different sterols and pentacyclic triterpenoids have been isolated and characterized from *Zea mays* [54]. A typical plant sterol profile from *A. thaliana* was characterized as 64% β-sitosterol, 11% campesterol, 6% stigmasterol, 3% isofucosterol, and 2% of brassicasterol [55]. β-sitosterol is also the most abundant sterol in the PM of tomato calli and broccoli roots [33,56], whereas stigmasterol predominates in the PM from oat roots [57]. Special attention is now being given not only to the structural roles of sterols, but also to their regulatory roles in the membrane. For example, β-sitosterol and campesterol play a significant role in the ordering of fatty acid chains in the membrane, which may affect membrane water and ion permeability, as well as the activity of membrane proteins. In addition, cholesterol screens negative charges and thereby decreases the membrane surface charge. It may contribute to denser packing of hydrocarbon chains in the gel phase and, thereby, increase membrane microviscosity, which could decrease permeability [58]. Changes in sterol composition of the membranes could be important for protecting the membrane from environmental stress [59].

Membrane glycerolipids have glycerol backbones with two fatty acid molecules bound to *sn-1* and *sn-2* and either a phosphorous (phospholipid) or sugar (glycolipid) molecule at position *sn-3*. Phosphatidylcholine (PC) and phosphatidylethanolamine (PE) are the dominant membrane phospholipids, which have been extensively studied (Table 4). Other phospholipids, including phosphatidic acid (PA) phosphatidylinositol (PI) and phosphatidylserine (PS) constitute the remainder of membrane phospholipids. Glycolipids, including monogalactosyldiacyloglycerol (MGDG), digalactosyldiacyloglycerol (DGDG), and sulfoquinovosyldiacylglycerol (SQDG), together with the phospholipid, phosphatidylglycerol (PG), make up the plastid membrane [60]. According to their molecular structure, glycerolipids can be divided into cylindrical shape bilayer forming lipids (e.g., PC, PA, PI, PG, PS, DGDG) and cone-shape non-bilayer forming lipids (e.g., PE, MGDG). Generally, bilayer lipids ensure the stability of membranes, whereas non-bilayer lipids are important for mediating proteolipid interactions and increasing morphological plasticity of lipid bilayers [61].

Fatty acid composition of membrane lipids is conserved across plant species with palmitic (16:0) (number of carbons in fatty acid chain: number of double bonds in fatty acid chain) and linoleic (18:2) accounting for the largest proportion (Table 5), followed by 18:3, 18:1, 18:0, and 16:1 fatty acids. Some rare fatty acids, for instance 14:0, 17:0, 20:0, have also been observed in a few studies (Table 5). The changes in ratio of saturated and unsaturated fatty acid components are implicated in plant responses to salt stress due to the ability to regulate membrane fluidity and permeability [62].

Although the main lipid classes are similar, there is a great diversity in their relative content in plant membranes across plant species, within the different organs or even between different organelles (Table 4 and Table 5). Salinity-induced alterations in membrane lipids, including the changes to content of lipids, their fatty acid components, bilayer to non-bilayer lipid ratio, and the activities of signaling lipids, could regulate the membrane fluidity and permeability, thereby altering the biological properties of plant membranes (Figure 1) [37,39,62,63]. These alterations could bring about the regulation of the proteins that participate in lipid biosynthesis and metabolism (Figure 1).

## 3. Alterations in Membrane Lipids in Response to Salt Stress

### 3.1. Total Membrane Lipid Content Changes under Salt Stress

Increased electrolyte leakage of membranes brought about by salinity has been reported in salt-sensitive plants, including roots of barley [26] and broccoli [56], leaves of tomato plants [70], and *Populus cathayana* [71], and was associated with a reduction in total lipid content, indicating a loss of membrane integrity. It was proposed that the stress-induced decrease in total membrane lipid content was a consequence of enhanced lipolysis and peroxidation, as well as an inhibition lipid biosynthesis pathways [72,73]. A reduction in lipid content under salt stress was also observed in a salt-sensitive cultivar of barley *Hordeum. vulgare* L. cv. Manel, while no change was observed in a salt-tolerant wild species *Hordeum. maritimum* [26]. This study also demonstrated that the ability to maintain lipid homeostasis under salt stress sustained cell expansion and growth of salt-stressed plants [26].

Salinity-induced increases in total membrane lipid content have been observed in several salt-tolerant plant species and algae [27,62,74,75,76] (Table 1). In these studies, the degree of changes in total membrane lipid content depended on the salt concentration and tissues studied. In the halophyte *Suaeda altissima*, highest total membrane lipid content was measured in aerial tissue of plants grown in 250 mM NaCl, a level two times more than that observed in plants grown under either lower or higher NaCl concentrations (1 mM and 750 mM). No significant changes were observed in total lipid content in the roots under salt stress [62].

While salt-induced alterations in membrane lipid content have been shown in many species, the specific lipid species that are altered are not the same. For example, elevated amounts of neutral lipids detected in *Catharanthus roseus* cell suspensions exposed to high salinity were responsible for the increased total membrane lipid content [25], while increased total membrane lipid content was attributed to enhanced glycerolipid abundance in salt-tolerant soybean under salt stress [77]. However, how these specific lipid species alter the physical properties of the membranes and affect the salt tolerance of the plants is unknown.

### 3.2. Changes in Membrane Phospholipids in Response to Salt Stress

Membrane phospholipids serve as structural and signaling molecules in plant cells [44,78] and reports have shown alterations in amount and species in plants subjected to salinity stress. Studies in maize have shown that the proportion of phospholipids in the root PM of a salt-tolerant genotype was approximately 1.7-fold higher when directly compared to a sensitive genotype [31], suggesting a positive correlation between phospholipid content and plant salt tolerance. Similar findings were also reported for dwarf cashew seedlings, with the proportion of phospholipids relative to total lipids in the salt tolerant line dramatically higher compared to the sensitive line [30].

Salt stress was shown to increase total phospholipid content in both salt-sensitive and salt-tolerant species, including in the PM of wheat roots [32], cell suspensions of *C. roseus* [25], root and cell suspensions of *Kosteletzkya virginica* [34], and epidermal bladder cells from *Mesembryanthemum crystallinum* [35] (Table 2). Nevertheless, a decline in phospholipid content were observed in several sensitive species [28,29]. In buffalo grass, phospholipid content decreased under salt stress in both the salt-sensitive line and the salt-tolerant line, although the overall reduction in the sensitive line was greater [29].

Among all the major phospholipids, PC and PE are the predominant components (together accounting for 15%–80%, Table 4) in membranes. Membrane PC significantly increased in callus culture of the halophyte *Spartina patens* [36], epidermal bladder cells of the halophyte *M. crystallinum* [35], roots of salt-tolerant *Plantago* cultivars [79], and salt-acclimated tomato calli [33,80]. In support of the positive role of PC in salt tolerance, the addition of choline, a key substrate for PC biosynthesis, improved the salt tolerance of wheat [81]. Increased PC was also seen in salt-sensitive species, such as in cell cultures of *C. roseus* [25] and *A. thaliana* [82]. Furthermore, simultaneous upregulation of choline kinase (CK) mRNA levels and enzyme activity was observed in leaf rosettes of *A. thaliana* in response to salt stress, which may contribute to the increased PC biosynthesis [82]. PC content also increased in membranes from both mesophyll and bundle sheath chloroplasts in salt-treated maize [39], and while phospholipids are not predominant components in chloroplast membranes, they can serve as precursor molecules for the synthesis of glycolipids. Reports have also shown a down regulation in PC content under salt stress, including in the PM of roots of maize and wheat [28,29,31,32]. This may indicate a possible damage to the integrity of these membranes.

In plants, the higher PC-to-PE ratio in membranes is often related to greater salt tolerance [39]. The increased PC level observed in *S. patens* callus cultures under salt stress led to a greater than two-fold change in the calculated ratio of bilayer to non-bilayer forming lipids [36]. It is suggested that the increased PC-to-PE ratio was correlated with faster callus proliferation [83]. Conversely, the decrease in PC to PE ratio of wheat in response to NaCl was assumed to result in a breakdown of membrane integrity and increased membrane permeability [28,84].

Although other phospholipids are less abundant than PC and PE, evidence has shown their changes are also crucial for plant salt tolerance. Upregulated PI and PS were found in root PM of salt-treated wheat [32], callus culture of *S. patens* [36], and roots from salt tolerant buffalo grass, while sensitive buffalo grass cultivars displayed a significant reduction in PI and PS levels [29]. It is assumed that owing to the higher amounts of negatively charged PI and PS, the root PM membrane of the grass would have a higher affinity for Ca^2+^ [29]. As a second messenger in stress signaling, Ca^2+^ can alleviate the negative effects of NaCl by activating Na^+^ efflux [85].

Phosphatidylglycerol (PG) plays a critical role in the function of photosystem II (PSII) in the thylakoid membranes. PG is believed to be the only lipid completely essential for oxygenic photosynthesis, with other plastidic lipids such as the galactolipids MGDG and DGDG, found to be replaceable with glucolipids [86], or in the case of SQDG, dispensable [87]. Increased PG under salt stress was observed in leaves of *Thellungiella halophila* [37], in epidermal bladder cells of the halophyte *M. crystallinum* [35], and in salt-tolerant buffalo grass [29]. As an integral component of photosynthetic membranes [88], it was speculated that the salt-induced increase in PG levels is important for maintaining the functioning of PSII by protecting the photosynthetic apparatus and stabilizing photosynthetic processes, such as ATP synthesis and light-harvesting complex II (LHCII). In support of this, a significant decline in PG in leaves from salt-stressed *Sulla carnosa* and *Sulla coronaria*, was speculated to result in ultrastructural damage of thylakoid membranes observed by transmission electron microscopy [40].

### 3.3. Changes in Membrane Glycolipids in Response to Salt Stress

Glycolipids, especially galactolipids, are important for photosynthesis, as they are the building blocks for the thylakoid membrane [89]. They are rich in unsaturated fatty acids, and alterations in fatty acid profiles (discussed in Section 3.5) of glycolipids has been shown to significantly affect membrane structural and functional properties of chloroplast membranes [37].

Salinity-induced reduction in glycolipids has been observed in rice, cucumber, and cowpea seedling leaves, resulting in a significant decrease in chlorophyll content [90,91,92]. In contrast, an increased galactolipid content was shown in leaves of a wild tobacco, which was suggested to contribute to the maintenance of photosynthetic membranes in the presence of NaCl [93]. Addition of Ca^2+^ stimulated total glycolipid content in salt-stressed cowpea leaves, which was suggested to positively affect membrane fluidity [94].

Salinity-induced decreases in MGDG were found in the leaves of mangrove [93], *S. carnosa* and *S. coronaria* [40], and callus culture of *C. roseus* [25]. The reduction in MGDG is a common response of plants to osmotic stress brought about by drought, salinity, or freezing (Table 3) [95,96]. This is thought to be a consequence of increased galactolipase and lipoxygenase activities, which are normally induced by environmental stress or cell senescence [97], and have a preference for the breakdown of MGDG [98]. Surprisingly, as a non-bilayer forming lipid, MGDG could form stable bilayer structures by its interaction with LHCII [99]. Thereby, salt-induced MGDG decline could result in serious disorder and dysfunction of photosynthetic membranes [100]. In other words, sufficient MGDG lipids are required for self-regulation of membranes in regards to its lipid (MGDG) to protein (LHCII) ratio to maintain a stable bilayer structure [101].

A decreased DGDG content with increasing NaCl concentration was observed in *C. roseus* cultured cell suspensions. Likewise, lowered DGDG levels were also found in *S. carnosa* and *S. coronaria* [40], and in membranes from leaves of *T. halophila* and *A. thaliana* [37], following salt-treatment. NaCl-induced decreases in DGDG is argued to be responsible for membrane structural disorder [93], instability of the membrane [102], and an alteration in membrane protein activity [103]. A similar mechanism was reported in *A. thaliana* in response to temperature stress, whereby the heat-induced reduction in DGDG hindered the membrane LHCII-macrodomain formation, reduced the stability of PSI, and shortened the lifetime of chlorophyll fluorescence measured by electrochromic absorbance transients [104].

Studies have shown that a change in the ratio of bilayer to non-bilayer forming glycolipids could give rise to physicochemical alterations to the membranes of plant cells thereby affecting their ability to tolerate salt stress [37,40,93]. In both *A. thaliana* and *T. halophila*, the DGDG-to-MGDG ratio decreased in leaves in response to salinity. However, the decrease in *A. thaliana* was greater, suggesting a better protection of membranes and photosynthetic apparatus in the more salt-tolerant *T. halophila* [37]. Similarly, the DGDG-to-MGDG ratio of mesophyll chloroplasts of maize was lower than that of the bundle sheath chloroplasts (relatively salt tolerant compared to mesophyll as they are less affected by reactive oxygen species and possess higher activities of antioxidant enzymes) under non-stressed conditions [105]. Interestingly, under salt stress, the DGDG-to-MGDG ratio increased greatly in the mesophyll chloroplasts, while that of the bundle sheath chloroplasts remained almost unchanged [39]. Changes in the DGDG-to-MGDG ratio in chloroplast membranes under salt stress can be brought about by changes in either of these lipid species; for instance, in Fabaceae [40], the decreased MGDG/DGDG ratio observed is mainly due to MGDG degradation, whereas in snow alga [76] it depended greatly on increased DGDG synthesis.

SQDG is the only glycolipid species found exclusively associated with photosynthetic membranes [106] and its presence is essential for PSII activity [107]. High levels of SQDG are often associated with salt tolerance in plants [108]. An elevated level of SQDG was detected in the leaves of several halophytes including *Aster tripolium*, *Sesuvium portulacastrum* [41], *T. halophila* [37], and *Crithmum maritimum* [27]. It was suggested that the ability of halophytes to increase SQDG levels in the membrane provided a more stable protein–lipid configuration [107,109], ensuring stability for photosynthesis in the presence of high salt [27]. In some glycophytes, such as *A. thaliana*, the level of SQDG also remarkably increased by 15.6% and 22.5% under NaCl treatments of 100 and 200 mM NaCl, respectively [37]. However, this result was not seen in all glycophytes, as in maize, the SQDG level in salt-treated plants was significantly lower compared to untreated plants [39]. Salinity treatment also caused a considerable decrease in SQDG levels in the chloroplast membrane from both *S. carnosa* and *S coronaria* leaves [40]. Although it is a less abundant glycolipid species, SQDG upregulation might be required for salt tolerance not only because it is closely associated with the photosynthetic apparatus, but also because it may play a role in signaling processes in plants during salt treatment. Seigneurin-Berny et al. [110] showed that SQDG may bind annexin (cellular proteins) in a Ca^2+^-dependent manner. The family of annexins are considered to play a role in the regulation of membrane organization, membrane fusion, and ion transport across membranes [111,112,113].

### 3.4. Changes in Membrane Sterols in Response to Salt Stress

Sterols are also structural components of cell membranes and ubiquitously present in plants. Membranes from salt-tolerant species/genotypes were found to be constitutively rich in sterols [33,34], whereas sterol content was seen to be lower in the most sensitive varieties [114]. Increases in total sterol content induced by NaCl treatment were found in salt-adapted tomato calli [33], in salt-tolerant wheat [31], and in the halophyte *K. irginica* [34]. In contrast, in non-tolerant species/genotypes, such as a sensitive wheat cultivar, the amount of sterol lipids was significantly reduced [32]. Based on this evidence, it was proposed that the ability to increase total sterol content under salt stress may be an important adaptive mechanism in salt tolerant species/genotypes [32]. Other studies have denoted that maintenance of a constant level of sterols in the membrane would be essential for plant salt tolerance [32,115].

In a study of salt-treated wheat, although NaCl had minor effects on total membrane sterol content, significant alterations could be detected in individual sterol species in the root tissue [28]. Cholesterol, stigmasterol, and brassicasterol were significantly increased by NaCl, resulting in a higher ratio of planar to non-planar sterols, which is thought to be beneficial for plant salt tolerance. Planar sterols integrate more readily into the liquid lipid phase of the membrane, while less-planar sterols disrupt membrane packing and can lead to a higher permeability of the membranes to Cl^-^ [116]. Salama and colleagues [32,117] also demonstrated an increased content of planar sterols, including campesterol and cholesterol, was related to improved salt tolerance in wheat caryopsis. Moreover, a reduction in the sitosterol was reported in the PM of salt-treated broccoli roots [56], which was associated with a reduction in the water permeability of the PM [118]. However, the changes in the ratio of more planar to less-planar membrane sterol appears to depend on the species and tissue studied, as well as the level of salt stress applied [25,116].

Apart from the total/individual sterol content, sterol/phospholipid ratios have also been implicated as a marker of membrane remodeling under salt stress. An elevation in the sterol to phospholipid ratio was observed in the halophytes *K. virginica* and *S. patens* [34,119], as well as in salt-tolerant wheat, tomato calli [31,33], and salt-resistant yeast [120], subjected to salt stress. Conversely, the sterol-to-phospholipid ratio decreased in the PM of a salt-sensitive maize cultivar [31]. The increased sterol to phospholipid ratio would likely result in a more rigid membrane, which may indirectly affect the permeability of Na^+^ and Cl^−^ through the regulation of the plasma membrane H^+^-ATPase hydrolytic and H^+^-pumping activities [33]. As shown by Grandmougin-Ferjani et al. [121], the activity of the plasma membrane H^+^-ATPase appeared to be remarkably sensitive to its sterol environment. In addition, several sterols, such as cholesterol and stigmasterol, were proven to have the ability to stimulate proton pumping, whereas sitosterol behaved as an inhibitor of pump activity [122].

### 3.5. Changes in Membrane Lipid Fatty Acids in Response to Salt Stress

Fatty acids are a common feature of complex lipids, defining the lipid species in relation to the fatty acid chain length, and the presence, number, and position of double bonds in the hydrocarbon chain. These variations can give rise to differences in the saturation of the fatty acid, with the greater number of double bonds increasing the unsaturation of the lipid. Saturated lipids generate liquid-order phases, and unsaturated lipids generate liquid-disordered phases, thereby the presence of fatty acid residues and their state of saturation can directly affect membrane fluidity [42]. Studies on fatty acids in salt-sensitive species, such as barley and maize, suggested they were more saturated; by contrast, fatty acids in salt-tolerant species were more unsaturated [26,123]. This could indicate that there is a relationship between the degree of unsaturation of membrane lipid fatty acids and plant salt tolerance but would need to be investigated in more detail. Plants including *S. patens* [36], maize [31], canola [38], buffalo grass [29], and wheat [32] showed an increased level of saturation or a decreased unsaturation of fatty acids in their membranes under salt stress. Unsaturated-to-saturated ratio of fatty acids decreased in roots of salt-treated canola [38], with the salt-tolerant cultivar showing a greater decline than that of the sensitive cultivar.

Reduced lipid unsaturation of fatty acids was assumed to be correlated with a decrease in membrane fluidity and it was thought that this would limit the uptake of Na^+^ and Cl^−^ across the PM by directly regulating transporters involved [28,29,119,124]. A reduction in membrane fluidity is also thought to be necessary to prevent leakage of the compatible solute glycerol, out of the cell and diffusion of potentially harmful ions into the cell, helping to maintain the cell osmotic pressure balance between the internal and external environments [125,126]. Furthermore, a reduction in the degree of unsaturation of lipid fatty acids is related to a decrease in the susceptibility of the membrane to oxidative damage, helping to protect membrane integrity [124].

Salama and Mansour [32] speculated that an increased degree of saturation in lipids would induce formation of a gel phase and result in a phase separation in the PM, impairing its proper functions. However, Bejaoui et al. [40] observed a significant decrease in the unsaturated-to-saturated ratio in fatty acids in salt-sensitive *S. carnosa* leaves, while no change was observed in the salt-tolerant genotypes. This suggested that the capacity to preserve a constant degree of unsaturation was related to salt tolerance [26]. In agreement with this statement, in mulberry leaves under saline conditions, the decomposition products of polyunsaturated fatty acids was higher in the salt-sensitive genotype, while no change was measured in the salt-tolerant genotype [127].

In contrast to the above results, the degree of unsaturation of fatty acids significantly increased in the PM from leaves of the halophyte *T. halophila* [37]. In agreement with this, research carried out to genetic engineer fatty acid desaturases also indicated the positive role of FA polyunsaturation in plant salt tolerance (Table A1). Moreover, the overexpression of ω-3 desaturases (*FAD3* or *FAD8*) in transgenic tobacco plants has been shown to increase the tolerance to both salt and drought stresses [128]. Allakhverdiev et al. [129] demonstrated that compared to wild-type cells, which contain polyunsaturated fatty acids, *desA*^−^/*desD*^−^ mutant strains of *Synechococcus* sp PCC 6803 (the *desA* and *desD* gene for the Δ12 and Δ6 desaturases had been inactivated by targeted mutagenesis), which only have monounsaturated fatty acids, were less tolerant to NaCl. It was speculated that the unsaturation of fatty acids in the membrane lipids protected the oxygen-evolving photosynthetic machinery against salt-induced inactivation and also activated the Na^+^/H^+^ exchanger salt-overly-sensitive 1 (SOS1), responsible for sodium extrusion from the cell, by means of enhanced fluidity of the membrane [56,130].

### 3.6. Changes in Membrane Neutral Lipids in Response to Salt Stress

Studies focusing specifically on the salt-induced changes in glycerolipids and sterols have ignored the importance of neutral lipids. Recent evidence has shed light on the role of triacylglycerols (TG), in plant stress tolerance. From these studies it was proposed that TG accumulation in vegetative tissues can serve as an energy reservoir under times of stress [131,132,133,134,135,136]. TG are primarily stored in lipid droplets (LD) or plastoglobules (chloroplast localized LD), along with steryl/wax esters. Increases in both LD and plastoglobule size and number have been observed under salt stress [35,40,137,138]. In leaves of the glycophyte *S. carnosa* [40], increased plastoglobule size and number under salt stress was linked to increased accumulation of TG lipids as a result of thylakoid galactolipid mobilization. It was hypothesized that this could be an intermediate step in the conversion of thylakoid fatty acids to phloem-mobile sucrose, recruiting membrane carbon for the normal growth of plants under salt stress. Interestingly, salt induced changes in cytoplasmic LD were observed in the epidermal bladder cells of the halophyte *M. crystallium* [35]. However, in contrast, in this study, decreased TG and downregulated TG biosynthesis under salt stress were correlated to a decrease in the number and size of cytosolic lipid droplets, while plastoglobuli remained unchanged. Downregulation of TG was proposed to be an adaptive feature of salt-tolerant plants and may provide precursor lipid molecules such as DAG and FA for phospholipid synthesis to increase membranes as the epidermal bladder cells expand rapidly due to massive increases in endopolyploidy. However, as only a limited number of studies have been reported on regulation of TG in vegetative tissues of plants under salt treatment, the precise function of TG during stress and the mechanisms behind the regulation need to be investigated further.

### 3.7. Membrane Lipids as Signaling Molecules in Plant Salt Tolerance

Although they are usually very low in abundance, signaling lipids can be synthesized and accumulate quickly, from pre-existing membrane lipids or intermediate products of lipid biosynthesis, in response to adverse environmental conditions [20]. A large number of studies have demonstrated an accumulation of the lipid secondary messenger phosphatidic acid (PA) in responses to multiple abiotic stresses, including salt stress [35,139,140]. PA can be either formed directly through hydrolysis of phospholipids by phospholipase D (PLD), (i.e., hydrolysis of PC, PE, PG, and PI) [95,141,142], or indirectly via sequential action of phospholipase C (PLC) and diacylglycerol (DAG) kinase [143,144,145]. Several studies have shown stimulated PLD activities in plants subjected to salt stress, which include cell cultures of *Chlamydomonas reinhardtii* [139], seedlings of *A. thaliana* [142], and tomato cell suspensions [146], resulting in increased PA levels. Moreover, stimulated PA synthesis was suppressed in PLDδ antisense transgenic *A. thaliana* [142], while knockout and overexpression of PLDα1 increased and decreased the salt sensitivity of *A. thaliana*, respectively [147]. Furthermore, an *A. thaliana* PLDα1 knockout mutant displayed decreased PA levels, and the double mutant *pldα1/pldδ* exhibited a higher sensitivity to salinity stress than either of the single mutants [146].

In contrast, PLD was reduced in tobacco pollen tubes under hyperosmotic stress [148], suggesting that responses could be stress-specific. Likewise, Darwish et al. [149] also demonstrated a short-term (30 min) NaCl stress in rice inhibited PLD activity, although PA levels increased in leaves, which was proposed to occur via the alternative PLC/DAG kinase pathway. In contrast, in epidermal bladder cells of *M. crystallinum*, increases in PA levels under salt-stress were attributed to activation of PLD as most of the transcripts in the PLC/DAG pathway were downregulated by salt treatment [35].

Worth mentioning is that PA can also influence the activity of membrane proteins [150]; for example, the activity of MAP65-1, which participates in the polymerization and bundling of microtubules, was stimulated by addition of exogenous PA in response to salt stress [151]. Furthermore, a study of maize seedlings showed PA stimulated the activity of the tonoplast H^+^-ATPase, which provides the driving force for Na^+^ accumulation into the vacuole [152].

Phosphatidylinositol bisphosphate (PIP_2_) is another typical membrane signaling phospholipid class responding to salinity stress. Increased PIP_2_ in plants subjected to salt or hyperosmotic stress has been observed in several plants, including in *A. thaliana* suspension cultures and seedlings [153], tobacco pollen and cell suspensions [148,154], rice leaves [149], and in the halophilic green alga *Dunaliella salina* [155]. Moreover, the secondary messenger inositol 1,4,5-trisphosphate (InsP_3_), hydrolyzed from PIP_2_ by PLC, is proposed to be involved in salt tolerance of Characean algae. InsP_3_ contributed to the initiation of action potentials (APs) in *Chara corallina* cells by releasing Ca^2+^ from internal stores, and propagating prolonged APs [156,157]. Supporting these findings was evidence for dramatically increased InsP_3_ levels in salt-treated *Daucus carota* cell culture and *Arabidopsis* plants [158,159], and the time frame of InsP_3_ accumulation was similar to that of salt-induced Ca^2+^ mobilization [158].

Recently, work on *A. thaliana* demonstrated that a plasma membrane glucuronosyltransferase, responsible for the synthesis of the glycosyl inositol phosphorylceramide (GIPC) sphingolipid, MOCA1, is required for salt-induced elevation of cytosolic free Ca^2+^ concentration [160]. It is hypothesized that Na^+^ binding to negatively charged GIPCs results in the depolarization of the membrane, which in turn activates Ca^2+^ influx channels. Cytoplasmic calcium is critical for the upregulation of the Na^+^/H^+^ exchanger involved in the removal of excess Na^+^. Furthermore, a Ca^2+^ mobilizing molecule, sphingosine-1-phosphate was demonstrated to be involved in the drought signal transduction pathway in *Commelina communis* linking the perception of abscisic acid to reductions in guard cell turgor [161]. However, despite these studies, not much is known about the binding of sphingolipids to ion channels or sphingolipid regulation in plants under ionic or osmotic stress.

## 4. Salt-Induced Changes to Lipid Metabolism

**Figure 2:**
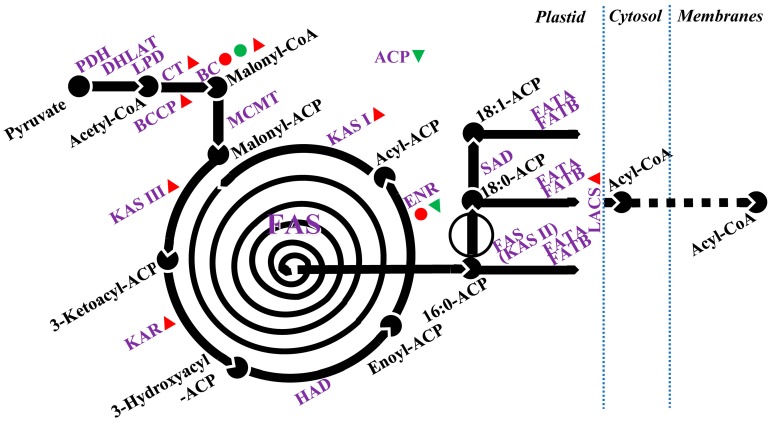
Pathway for fatty acid synthesis, elongation, and export. Information adapted from Li-Beisson et al. [163]. Partial intermediate products are not shown. Dotted blue line represents the division of different subcellular components. The symbols ●, ●, ▲ and ▼ indicate upregulated proteins, downregulated proteins, upregulated transcripts, and down regulated transcripts, respectively, by salt stress, reported in the publications listed in Table 6. De novo fatty acid synthesis begins from an acetyl-coenzyme A (Acetyl-CoA) generated from a pyruvate catalyzed by pyruvate dehydrogenase, dihydrolipoyllysine-residue acetyltransferase (DHLAT), and dihydrolipoamide dehydrogenase (LPD). Biotin carboxylase catalyzes acetyl-CoA to produce malonyl-CoA. Fatty acids are elongated by sequential condensation of two-carbon units by enzymes of the fatty acid synthase complex. To generate a C16 fatty acid, the cycle needs to be repeated seven times and several enzymes are involved, including ketoacyl-ACP synthase (KAS) I, II, and III, ketoacyl-ACP reductase (KAR), hydroxyacyl-ACP dehydrase (HAD), and enoyl-ACP reductase (ENR); acyl carrier protein (ACP) is a cofactor in all reactions. Fatty acyl thioesterase (FAT), stearoyl-ACP desaturase (SAD), and long-chain acyl-CoA synthetase (LACS) are required for fatty acid elongation and transport to other organelles.

**Figure 3:**
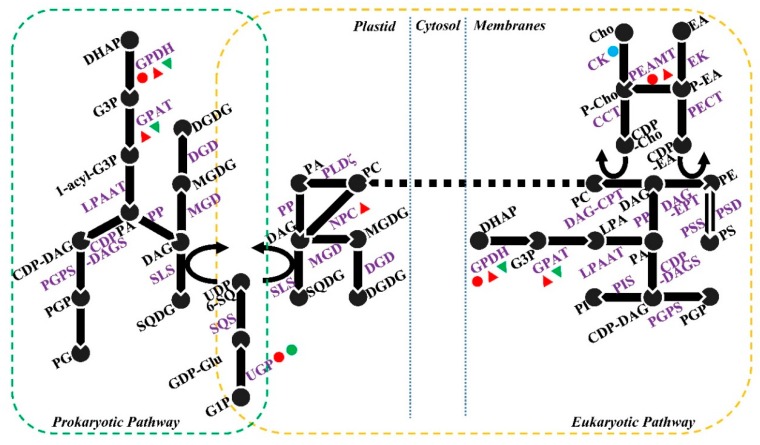
Proteins involved in glycerolipid synthesis. Partial intermediate products are not shown. Information adapted from Li-Beisson et al. [163]. Dotted blue line represents the division of different subcellular components. The symbols ●, ●, ●, ▲ and ▼ indicate salinity-induced upregulated proteins, downregulated proteins, unchanged proteins, upregulated transcripts, and downregulated transcripts, respectively, reported in the publications listed in Table 6. Partial intermediate products are not shown. Plant prokaryotic lipid synthesis occurs exclusively and entirely inside the plastid. In prokaryotic pathways, glycerol 3-phosphate (G3P) is firstly acylated with an 18:1 ACP at sn-1 position by glycerol-3-phosphate acyltransferase, then the product is acylated with 16:0 ACP at sn-2 position by lysophosphatidic acid acyltransferase (LPPAT) to produce a phosphatidic acid (PA). PA is then either dephosphorylated to produce DAG by PA phosphatase or converted to PGP utilizing CDP-DAG synthase (CDP-DAGS) and phosphatidylglycerophosphate synthase (PGPS). PGP phosphatase is used for PGP dephosphorylation. Two transferase, monogalactosyldiacylglycerol transferase (MGDGS) and digalactoslydiacylglycerol synthase (DGDGS), are involved in the formation of MGDG and DGDG from DAG. The formation of SQDG needs the sulphite transfer to DAG by sulfolipid synthase (SLS) from UDP 6-sulfoquinovosyl, which is produced by the condensation of UPD-glucose with sulphite by UDP-sulfoquinovose synthase (SQS). UDP-glucose pyrophosphorylase (UGP) is responsible for the conversion of glucose-1-phosphate to UDP-glucose. Eukaryotic phospholipid is also synthesized from G3P to produce DAG and CDP-DAG through PA using the same enzymes as prokaryotic lipid synthesis, although it uses acyl-CoA for the sequential acylation reactions. In addition to PGP formation, PI can also be produced from CDP-DAG by phosphatidylinositol synthase (PIS). DAG are then combined with CDP-choline and CDP-ethanolamine to produce PC and PE, respectively. These proteins can be derived from free choline (ethanolamine) by successive enzymatic reactions of choline (ethanolamine) kinase (CK and EK) and CTP: Phosphorylcholine (phosphorylethanolamine) cytidylyltransferase. Phosphoethanlamine can be methylated to phosphocholine by phosphoethanolamine *N*-methyltransferas (PEAMT) and converted to PC. PE is a substrate for PS biosynthesis by phosphatydylserine synthase PSS, whereas PS is converted to PE by phosphatidylserine decarboxylase (PSD).

Lipids are important structural, storage, and signaling molecules of the plant cell, being principal components of plant membranes and the cuticle, as well as making up a large component of seeds [162]. In the model plant *A. thaliana*, more than 600 genes encode the proteins involved in at least 120 enzymatic reactions of the plant lipid biosynthesis pathways [163]. Despite their critical role in the plant, lipid metabolism has been mostly ignored in studies looking at effects of abiotic stress, with the exception of cold and heat stress [131,164]. Only a few reports have focused on the effects of salt stress on cellular lipid homeostasis including fatty acid synthesis, elongation, and export (Figure 2) and glycerolipid synthesis (Figure 3).

Microarray hybridization studies have revealed alterations in genes encoding proteins involved in fatty acid synthesis and elongation under salt stress in *A. thaliana* and its highly salt-tolerant relative *T. halophila* [165]. In both species, the genes encoding acyl carrier proteins (ACP1, ACP4), enoyl-ACP reductase (MOD1), and dihydrolipoyllysine-residue acetyltransferase (LTA2) were downregulated [165]; whereas increased transcription of biotin carboxylase (CAC2), long-chain acyl-CoA synthetase (LACS2), and ketoacyl-ACP synthase (KAS I and KAS III) were found only in *T. halophila* [165]. Salinity stress of salt sensitive alfalfa root tissue triggered a down regulation in biotin carboxylase as observed by 2-D electrophoresis [166]. In contrast, upregulated biotin carboxylase was observed by 2-D electrophoresis in salt-treated cucumber leaf tissue [167]. Evidence appears to support the view that roots are less affected by NaCl than leaves in salt sensitive species/cultures [168]. Thereby, the upregulation of biotin carboxylase abundance in cucumber leaves may be related to a stimulated de novo fatty acid synthesis under salt stress.

In another proteomic study, ENR catalyze, the final enzyme in the de novo fatty acid biosynthesis cycle, was upregulated up to 42% in young rice panicles upon exposure to salinity [169]. Increased ENR abundance observed in this study may result in enhanced fatty acid synthesis that would support increased biosynthesis of cellular membranes to replace those damaged under salinity stress.

Glycerol-3-phosphate dehydrogenase (GPDH) is a multifunctional protein that is important for glycerolipid synthesis. It acts by dehydrogenating dihydroxyacetonephosphate (DHAP) to G3P. The level of *G3pdh* gene expression in the green alga *D. salina* was shown to be negatively correlated to NaCl concentration under short-term salt stress [170]. However, accumulation of glycerol was observed with increased salinity, suggesting there may be other GPDH isozymes that regulated glycerol metabolism under saline conditions [170]. In rice, three GPDHs (*OsGAPC*1-3) were found to be responsive to NaCl. Overexpression of the most highly salt-induced transcript, *OsGAPC*3, conferred tolerance to salinity [171], when compared to the wild type (WT) grown under similar conditions. Further evidence for a role of this protein under salt stress comes from experiments in which introduction of an oyster mushroom GPDH enhanced the tolerance of potato cell cultures against salt loading [172]. Zhang et al. [171] demonstrated the improved salt tolerance of rice overexpressing GPDH was via regulation of hydrogen peroxide levels; whether it is related to the regulation of lipid metabolism or membrane properties remains unknown.

Glycerol-3-phosphate acyltransferase, GPAT, is a rate-limiting enzyme in the glycerolipid synthesis pathway. Accumulation of mRNA for GPAT in leaves from salt-treated *Suaeda salsa* was observed by real-time PCR [178]. Overexpression of *SsGPAT* in *A. thaliana* protected against the salt-induced loss of chlorophyll content and helped to maintain both efficiency of PSII, PSI oxidoreductive activity and levels of unsaturated fatty acid content in the presence of salt. In contrast, *SsGPAT* t-DNA insertion mutants exposed to salinity stress showed decreases in all these factors [178]. These results suggested that unsaturation of PG could function to protect the thylakoid membrane under salt-stress by alleviating the photoinhibition of PSII and PSI [178]. Similarly, overexpression of chloroplastic GPAT in tomato increased unsaturation of PG in the thylakoid membrane, conferring transgenic plants a higher activity of chloroplastic antioxidant enzymes, lower content of reactive oxygen species, and less ion leakage under salt-stress conditions [188]. In another study, a key enzyme involved in the biosynthesis of galactolipids, monogalactosyldiacylglycerol synthase, MGD, was also found to be responsible for the protection of photosynthetic membranes during salt stress [93]. Overexpression of rice MGD in tobacco improved salt tolerance in transgenic plants by protecting thylakoid membranes and maintaining higher chlorophyll levels compared to untreated WT plants [93].

UDP-glucose pyrophosphorylase (UGP) is the committed enzyme for the first step of sulfolipid biosynthesis. *A. thaliana ugp3* mutants did not accumulate SQDG in leaves [189]. The regulation of UGP by salt has been seen in various species/varieties with contrasting results. For instance, UGP levels were decreased by salinity in rice roots [182], whereas the protein was accumulated in tomato leaves [180], and exclusively upregulated in roots of tolerant barley compared to a sensitive line [181]. However, UGP has mostly been studied as a regulator of sucrose and polysaccharides in plant salt-tolerance [181,190,191], and studies have largely ignored its role in lipid metabolism and regulation of SQDG under salt stress. As we discussed in Section 3.3, SQDG, which is exclusively located in the inner envelope of the chloroplast and is essential for PSII activity, was differently regulated by salt stress in halophytes vs. glycophytes [37,39]. This regulation may be due to the differences in UGP levels or activity, since UGP is the sulphite donor limiting SQDG production.

Phosphoethanolamine *N*-methyltransferase (PEAMT) participates in glycerophospholid metabolism. PEAMT transcripts have been shown to be strongly upregulated by salinity stress in sugar beet cell cultures [184], in maize roots, stems, and leaves [183], and in barley shoots [192] and roots [193]. Overexpression of a maize PEAMT improved the salt tolerance of transgenic *Arabidopsis* [183]. Conversely, an *Arabidopsis* PEAMT null mutant was hypersensitive to salt stress [194]. PEAMT is widely believed to be a key enzyme involved in plant choline biosynthesis, catalysing the *N*-methylation of phosphoethanolamine to convert phosphoethanoloamine to phosphocholine. Choline can be used by plants to produce glycine betaine, which has been shown to act as an osmoprotectant [32]. A higher content of glycine betaine is correlated with enhanced salt tolerance [195,196]. However, the role of PEAMT in membrane lipid metabolism has been overlooked. PC is the dominant membrane lipid constituent [197], and this phospholipid can also be converted to the important signaling molecule PA and free choline by phospholipase D (PLD). The salt-induced regulation of PC is widely studied (discussed in Section 3.2); however, the relationship between PEAMT and PC accumulation in plants under salt stress remains unclear.

Unlike PC, which can be synthesized via alternative pathways, phospahtidylserine (PS) is only produced from decarboxylation of PE, so the changes to PS (discussed in Section 3.2) in plants exposed to salinity stress should largely rely on phosphatidylserine synthase (PSD) and phosphatidylserine decarboxylase (PSS). Nevertheless, to the best of our knowledge, there have been no studies that have investigated this pathway under salt stress. The only evidence of increased activity of PSS and non-regulated PSD was observed in root cells of oat during drought acclimation, where it was shown the levels of PE are increased relative to PC resulting in increased drought tolerance by increasing the fluidity of the membrane [198].

Changes in the content of many membrane lipid species and/or the degree of saturation of fatty acids have been shown in a large number of plants upon salt stress (Section 3), however, how the enzymes that are involved in their biosynthesis and metabolism are regulated have been much less studied. Using the fragmentary knowledge that has been published to date, it is difficult to get a complete picture and understanding of the salt-induced membrane lipidome in plants without more work in this area.

## 5. Challenges and Perspectives

Development of crops with the ability to withstand salinity stress and grow on marginalized land represents a key driver to ensure global food security to an increasing world population. For this goal to be reached, there is an urgent need for a more comprehensive understanding of the consequences of salinity and the mechanisms of tolerance in plants. Studies at the molecular level have greatly expanded the depth of our knowledge in this area [199,200,201,202,203,204]. However, the majority of this research focuses on the proteins involved in signaling or ion transport, and there is limited work in the area of plant lipids. As a subgroup of the metabolome, the study of the plant lipidome could provide a source of biomarkers for salinity. Over the last three decades, technological advancement in analytical approaches, especially the improvement in MS-based technologies, have enabled substantial improvement in identifying and quantifying the multitude of lipid species in a cell, and further efforts in this area could greatly enrich our knowledge of plant stress responses at the metabolome level.

Furthermore, a means to dig deeper into the membrane lipidome is to understand changes occurring in membrane remodeling for particular organelles, which could provide important insight into the roles of different organelles during times of stress and help identify key lipid signaling molecules. Evidence supporting this is the increased level of PIP_2_ measured in microsomes from *Galdieria sulphuraria* under salt stress while levels in the plasma membrane were shown to be decreased, suggesting lipids could be regulated differentially in different organelles [205]. Purification of the subcellular membrane to the highest possible level prior to lipid analysis is desirable for in-depth lipid analysis and may help to identify new species that are too low in abundance when total membrane lipid analysis is carried out.

## Figures and Tables

**Table 1:** Alterations in total lipid content under salt treatment.

Species	Tissue	Salt Tolerance Level ^a^	NaCl Treatment	Treatment Duration	LC_salt-treated_/LC_c__ontrol_	Ref.
*Catharanthus roseus*	cell culture	salt-sensitive	50 mM	1 week	1.5 *	[25]
			50 mM	32 weeks	1.5 *	
			100 mM	1 week	1.2 *	
*Hordeum vulgare*	roots	salt-tolerant	50 mM	4.3 weeks	0.7 *	[26]
			100 mM	4.3 weeks	0.7 *	
			150 mM	4.3 weeks	0.6 *	
			200 mM	4.3 weeks	0.4 *	
*Hordeum maritimum*	roots	salt-tolerant	50 mM	4.3 weeks	1.4 *	[26]
			100 mM	4.3 weeks	1.1	
			150 mM	4.3 weeks	0.9	
			200 mM	4.3 weeks	0.7 *	
*Crithmum maritimum*	leaves	halophyte	50 mM	5 weeks	1.3 *	[27]
			100 mM	5 weeks	1.3 *	
*Crithmum maritimum*	leaves	halophyte	200 mM	5 weeks	0.9 *	[27]

^a^ Salt-sensitive and salt-tolerant in this column refer to glycophytes’ salt tolerance level. *: Lipid content (LC) of salt-treated samples is significantly different from control.

**Table 2:** Alterations in phospholipid (PL) content under salt treatment ^a^.

Lipid Class	Species	Tissue	Salt Tolerant Level ^b^	Membrane Class	NaCl Treatment	Treatment Duration	LC_salt__-treated_/LC_control_	Ref.
total PL	*Triticum aestivum*	roots	salt-sensitive	PM	100 mM	1 week	0.7 *	[28]
	*Buchloe dactyloides*	roots	salt-sensitive	PM	100 mM	4 days	0.7 *	[29]
	*Anacardium occidentale*	seedling	salt-sensitive	PM	8 dS m^−1 c^	4 weeks	1.9 *	[30]
	*Catharanthus roseus*	cell culture	salt-sensitive	total	50 mM	1 week	1.0 *	[25]
	*Catharanthus roseus*	cell culture	salt-sensitive	total	50 mM	32 weeks	1.2 *	[25]
	*Catharanthus roseus*	cell culture	salt-sensitive	total	100 mM	1 week	1.9 *	[25]
	*Zea mays*	roots	salt-sensitive	PM	150 mM	2.1 weeks	2.6 ^#^	[31]
	*Triticum sativum*	roots	salt-sensitive	PM	150 mM	3 weeks	1.7 *	[32]
	*Anacardium occidentale*	seedling	salt-tolerant	PM	8 dS m^−1 c^	4 weeks	1.1	[30]
	*Buchloe dactyloides*	roots	salt-tolerant	PM	100 mM	4 days	0.9 *	[29]
	*Lycopersicon esculentum*	calli	salt-tolerant	PM	50 mM	24 weeks	1.1 ^#^	[33]
	*Lycopersicon esculentum*	calli	salt-tolerant	PM	100 mM	24 weeks	1.5 ^#^	[33]
	*Kosteletzkya virginica*	roots	halophyte	PM	85 mol m^−3^	2 weeks	2.3	[34]
	*Kosteletzkya virginica*	cell culture	halophyte	PM	85 mol m^−3^	2 weeks	3.2 *	[34]
	*Mesembryanthemum crystallinum*	epidermal bladder cells	halophyte	total	200 mM	2 weeks	1.5 *	[35]
	*Spartina patens*	cell culture	halophyte	PM	170 mM	10 weeks	1.2	[36]
	*Spartina patens*	cell culture	halophyte	PM	340 mM	10 weeks	1.6	[36]
	*Zea mays*	roots	salt-sensitive	PM	150 mM	2.1 weeks	0.6 ^#^	[31]
PC	*Triticum aestivum*	roots	salt-sensitive	PM	100 mM	1 week	0.9 *	[28]
	*Catharanthus roseus*	cell culture	salt-sensitive	total	50 mM	1 week	1.0	[25]
	*Catharanthus roseus*	cell culture	salt-sensitive	total	50 mM	32 weeks	1.1 *	[25]
	*Catharanthus roseus*	cell culture	salt-sensitive	total	100 mM	1 week	1.6 *	[25]
	*Zea mays*	roots	salt-sensitive	PM	150 mM	2.1 weeks	0.8 ^#^	[31]
	*Triticum sativum*	roots	salt-sensitive	PM	150 mM	3 weeks	0.7 *	[32]
	*Buchloe dactyloides*	roots	salt-sensitive	PM	100 mM	4 days	0.4 *	[29]
	*Lycopersicon esculentum*	calli	salt-tolerant	PM	50 mM	24 weeks	1.0 ^#^	[33]
	*Lycopersicon esculentum*	calli	salt-tolerant	PM	100 mM	24 weeks	1.4 ^#^	[33]
	*Zea mays*	roots	salt-tolerant	PM	150 mM	2.1 weeks	0.4 ^#^	[31]
	*Buchloe dactyloides*	roots	salt-tolerant	PM	100 mM	4 days	1.5 *	[29]
	*Spartina patens*	cell culture	halophyte	PM	170 mM	10 weeks	≈1.1	[36]
	*Spartina patens*	cell culture	halophyte	PM	340 mM	10 weeks	≈1.8 *	[36]
	*Mesembryanthemum crystallinum*	epidermal bladder cells	halophyte	total	200 mM	2 weeks	2.2 *	[35]
PE	*Triticum aestivum*	roots	salt-sensitive	PM	100 mM	1 week	1.0	[28]
	*Catharanthus roseus*	cell culture	salt-sensitive	total	50 mM	1 week	1.0	[25]
	*Catharanthus roseus*	cell culture	salt-sensitive	total	50 mM	32 weeks	1.1	[25]
	*Buchloe dactyloides*	roots	salt-sensitive	PM	100 mM	4 days	1.0	[29]
	*Buchloe dactyloides*	roots	salt-sensitive	PM	100 mM	1 week	1.9 *	[29]
	*Zea mays*	roots	salt-sensitive	PM	150 mM	2.1 weeks	1.0 ^#^	[31]
	*Triticum sativum*	roots	salt-sensitive	PM	150 mM	3 weeks	1.8 *	[32]
	*Lycopersicon esculentum*	calli	salt-tolerant	PM	50 mM	24 weeks	1.2 ^#^	[33]
	*Lycopersicon esculentum*	calli	salt-tolerant	PM	100 mM	24 weeks	1.4 ^#^	[33]
	*Zea mays*	roots	salt-tolerant	PM	150 mM	2.1 weeks	0.6 ^#^	[31]
	*Buchloe dactyloides*	roots	salt-sensitive	PM	100 mM	4 days	0.9	[29]
	*Mesembryanthemum crystallinum*	epidermal bladder cells	halophyte	total	200 mM	2 weeks	1.1	[35]
	*Spartina patens*	cell culture	halophyte	PM	170 mM	10 weeks	≈0.7	[36]
	*Spartina patens*	cell culture	halophyte	PM	340 mM	10 weeks	≈1.0	[36]
PC/PE	*Catharanthus roseus*	cell culture	salt-sensitive	total	50 mM	1 week	1.0 ^#^	[25]
	*Catharanthus roseus*	cell culture	salt-sensitive	total	50 mM	32 weeks	1.1 ^#^	[25]
	*Catharanthus roseus*	cell culture	salt-sensitive	total	100 mM	1 week	0.8 ^#^	[25]
	*Triticum sativum*	roots	salt-sensitive	PM	100 mM	1 week	0.8	[28]
	*Zea mays*	roots	salt-sensitive	PM	150 mM	2.1 weeks	0.8 ^#^	[31]
	*Triticum sativum*	roots	salt-sensitive	PM	150 mM	3 weeks	0.4 *	[32]
	*Zea mays*	roots	salt-tolerant	PM	150 mM	2.1 weeks	0.6 ^#^	[31]
PS	*Zea mays*	roots	salt-sensitive	PM	150 mM	2.1 weeks	1.4 ^#^	[31]
	*Buchloe dactyloides*	roots	salt-sensitive	PM	100 mM	4 days	0.4 *	[29]
	*Triticum sativum*	roots	salt-sensitive	PM	150 mM	3 weeks	2.2 *	[32]
	*Zea mays*	roots	salt-tolerant	PM	150 mM	2.1 weeks	0.7 ^#^	[31]
	*Buchloe dactyloides*	roots	salt-tolerant	PM	100 mM	4 days	1.3 *	[29]
	*Spartina patens*	cell culture	halophyte	PM	170 mM	10 weeks	≈7.2 *	[36]
	*Spartina patens*	cell culture	halophyte	PM	340 mM	10 weeks	≈7.8 *	[36]
PI	*Zea mays*	roots	salt-sensitive	PM	150 mM	2.1 weeks	1.4 ^#^	[31]
	*Buchloe dactyloides*	roots	salt-sensitive	PM	100 mM	4 days	0.5 *	[29]
	*Triticum aestivum*	roots	salt-sensitive	PM	100 mM	1 week	1.1	[28]
	*Triticum sativum*	roots	salt-sensitive	PM	150 mM	3 weeks	1.5 *	[32]
	*Zea mays*	roots	salt-tolerant	PM	150 mM	2.1 weeks	0.6^#^	[31]
	*Buchloe dactyloides*	roots	salt-tolerant	PM	100 mM	4 days	1.9 *	[29]
	*Spartina patens*	cell culture	halophyte	PM	170 mM	10 weeks	≈3.5 *	[36]
	*Spartina patens*	cell culture	halophyte	PM	340 mM	10 weeks	≈2.4 *	[36]
PG	*Triticum aestivum*	roots	salt-sensitive	PM	100 mM	1 week	1.0	[28]
	*Zea mays*	roots	salt-sensitive	PM	150 mM	2.1 weeks	0.9^#^	[31]
	*Triticum sativum*	roots	salt-sensitive	PM	150 mM	3 weeks	0.8	[32]
	*Buchloe dactyloides*	roots	salt-sensitive	PM	100 mM	4 days	1.4	[29]
	*Arabidopsis thaliana*	leaves	salt-sensitive	total	100 mM	5 days	0.7 *	[37]
	*Arabidopsis thaliana*	leaves	salt-sensitive	total	200 mM	5 days	0.5 *	[37]
	*Zea mays*	roots	salt-tolerant	PM	150 mM	2.1 weeks	2.8 ^#^	[31]
	*Buchloe dactyloides*	roots	salt-tolerant	PM	100 mM	4 days	2.2 *	[29]
	*Thellungiella halophila*	leaves	halophyte	total	100 mM	5 days	1.0	[37]
	*Thellungiella halophila*	leaves	halophyte	total	200 mM	5 days	1.3 *	[37]
	*Thellungiella halophila*	leaves	halophyte	total	300 mM	5 days	1.3 *	[37]
	*Mesembryanthemum crystallinum*	epidermal bladder cells	halophyte	total	200 mM	2 weeks	1.5 *	[35]
PA	*Triticum aestivum*	roots	salt-sensitive	PM	100 mM	1 week	1.3	[28]
	*Zea mays*	roots	salt-sensitive	PM	150 mM	2.1 weeks	0.7 ^#^	[31]
	*Triticum sativum*	roots	salt-sensitive	PM	150 mM	3 weeks	0.7	[32]
	*Lycopersicon esculentum*	calli	salt-tolerant	PM	50 mM	24 weeks	1.2 ^#^	[33]
	*Lycopersicon esculentum*	calli	salt-tolerant	PM	100 mM	24 weeks	1.7 ^#^	[33]
	*Zea mays*	roots	salt-tolerant	PM	150 mM	2.1 weeks	0.7 ^#^	[31]

^a^ Abbreviations: PL: Phospholipids; PC: Phosphatidylcholine; PE: Phosphatidylethanolamine; PS: Phosphatidylserine; PI: Phosphatidylinositol; PG: Phosphatidylglycerol; PA: Phosphatidic acid; PM: Plasma membrane. ^b^ Salt-sensitive and salt-tolerant in this column refer to glycophytes’ salt tolerance level. ^c^ NaCl solutions electrical conductivities of 8 dS m^−1^. * Lipid content (LC) of salt-treated samples is significantly different from control. ^#^: Significance not specified. ≈: Estimated based on graphs.

**Table 3:** Alterations in glycolipid (GL) contents under salt treatment ^a^.

Lipid Class.	Species	Tissue	Salt Tolerant Level ^b^	Membrane Class	NaCl Treatment	Treatment Duration	LC_salt__-treated_/LC_control_	Ref.
total GL	*Triticum aestivum*	roots	salt-sensitive	PM	100 mM	1 week	1.1	[28]
	*Zea mays*	roots	salt-sensitive	PM	150 mM	2.1 weeks	0.8 ^#^	[31]
	*Brassica Napus*	roots	salt-sensitive	PM	50 mM	4.3 weeks	0.8 ^# c^	[38]
	*Brassica Napus*	roots	salt-sensitive	PM	100 mM	4.3 weeks	0.8 ^# c^	[38]
	*Brassica Napus*	roots	salt-sensitive	PM	150 mM	4.3 weeks	0.7 ^# c^	[38]
	*Brassica Napus*	roots	salt-sensitive	PM	200mM	4.3 weeks	0.7 ^# c^	[38]
	*Zea mays*	roots	salt-tolerant	PM	150 mM	2.1 weeks	0.6 ^#^	[31]
	*Brassica Napus*	roots	salt-tolerant	PM	50 mM	4.3 weeks	0.7 ^# d^	[38]
	*Brassica Napus*	roots	salt-tolerant	PM	100 mM	4.3 weeks	0.7 ^# d^	[38]
	*Brassica Napus*	roots	salt-tolerant	PM	150 mM	4.3 weeks	0.7 ^# d^	[38]
	*Brassica Napus*	roots	salt-tolerant	PM	200mM	4.3 weeks	0.6 ^# d^	[38]
	*Spartina patens*	cell culture	halophyte	PM	170 mM	10 weeks	1.2	[36]
	*Spartina patens*	cell culture	halophyte	PM	340 mM	10 weeks	1.1	[36]
MGDG	*Catharanthus roseus*	cell culture	salt-sensitive	total	50 mM	1 week	0.9	[25]
	*Catharanthus roseus*	cell culture	salt-sensitive	total	50 mM	32 weeks	0.8 *	[25]
	*Catharanthus roseus*	cell culture	salt-sensitive	total	100 mM	1 week	0.6 *	[25]
	*Arabidopsis thaliana*	leaves	salt-sensitive	total	100 mM	5 days	1.1	[37]
	*Arabidopsis thaliana*	leaves	salt-sensitive	total	200 mM	5 days	1.2 *	[37]
	*Zea mays*	mesophyll	salt-sensitive ^e^	chloroplasts	3%	5 days	≈0.6 *	[39]
	*Sulla carnosa*	leaves	salt-sensitive	total	200 mM	2.9 weeks	≈0.3	[40]
	*Sulla coronaria*	leaves	salt-sensitive	total	200 mM	2.9 weeks	≈0.5 *	[40]
	*Zea mays*	bundle sheath	salt-tolerant ^e^	chloroplasts	3%	5 days	≈1.0	[39]
	*Thellungiella halophila*	leaves	halophyte	total	100 mM	5 days	1.0	[37]
	*Thellungiella halophila*	leaves	halophyte	total	200 mM	5 days	0.9 *	[37]
	*Thellungiella halophila*	leaves	halophyte	total	300 mM	5 days	0.9 *	[37]
	*Mesembryanthemum crystallinum*	epidermal bladder cells	halophyte	total	200 mM	2 weeks	1.2	[35]
	*Crithmum maritimum*	leaves	halophyte	total	50 mM	5 weeks	1.3 *	[27]
	*Crithmum maritimum*	leaves	halophyte	total	100 mM	5 weeks	1.2	[27]
	*Crithmum maritimum*	leaves	halophyte	total	200 mM	5 weeks	0.8	[27]
DGDG	*Catharanthus roseus*	cell culture	salt-sensitive	total	50 mM	1 week	1.3 *	[25]
	*Catharanthus roseus*	cell culture	salt-sensitive	total	50 mM	32 weeks	1.3 *	[25]
	*Catharanthus roseus*	cell culture	salt-sensitive	total	100 mM	1 week	0.7 *	[25]
	*Arabidopsis thaliana*	leaves	salt-sensitive	total	100 mM	5 days	0.8 *	[37]
	*Arabidopsis thaliana*	leaves	salt-sensitive	total	200 mM	5 days	0.7 *	[37]
	*Zea mays*	mesophyll	salt-sensitive ^e^	chloroplasts	3%	5 days	≈1.1	[39]
	*Sulla carnosa*	leaves	salt-sensitive	total	200 mM	2.9 weeks	≈0.3 *	[40]
	*Sulla coronaria*	leaves	salt-sensitive	total	200 mM	2.9 weeks	≈0.5 *	[40]
	*Zea mays*	bundle sheath	salt-tolerant ^e^	chloroplasts	3%	5 days	1.09	[39]
	*Thellungiella halophila*	leaves	halophyte	total	100 mM	5 days	0.8 *	[37]
	*Thellungiella halophila*	leaves	halophyte	total	200 mM	5 days	0.6 *	[37]
	*Thellungiella halophila*	leaves	halophyte	total	300 mM	5 days	0.6 *	[37]
	*Mesembryanthemum crystallinum*	epidermal bladder cells	halophyte	total	200 mM	2 weeks	1.2	[35]
	*Crithmum maritimum*	leaves	halophyte	total	50 mM	5 weeks	1.4	[27]
	*Crithmum maritimum*	leaves	halophyte	total	100 mM	5 weeks	1.3	[27]
	*Crithmum maritimum*	leaves	halophyte	total	200 mM	5 weeks	0.7 *	[27]
SQDG	*Catharanthus roseus*	cell culture	salt-sensitive	total	50 mM	1 week	1.3	[25]
	*Catharanthus roseus*	cell culture	salt-sensitive	total	50 mM	32 weeks	2.9 *	[25]
	*Catharanthus roseus*	cell culture	salt-sensitive	total	100 mM	1 week	2.3 *	[25]
	*Arabidopsis thaliana*	leaves	salt-sensitive	total	100 mM	5 days	1.2 *	[37]
	*Arabidopsis thaliana*	leaves	salt-sensitive	total	200 mM	5 days	1.2 *	[37]
	*Zea mays*	mesophyll	salt-sensitive ^e^	chloroplasts	3%	5 days	0.6 *	[39]
	*Sulla carnosa*	leaves	salt-sensitive	total	200 mM	2.9 weeks	≈0.4 *	[40]
	*Sulla coronaria*	leaves	salt-sensitive	total	200 mM	2.9 weeks	≈0.6 *	[40]
	*Zea mays*	bundle sheath	salt-tolerant ^e^	chloroplasts	3%	5 days	0.7 *	[39]
	*Crithmum maritimum*	leaves	halophyte	total	50 mM	5 weeks	3.3 *	[27]
	*Crithmum maritimum*	leaves	halophyte	total	100 mM	5 weeks	2.8 *	[27]
	*Crithmum maritimum*	leaves	halophyte	total	200 mM	5 weeks	2.6	[27]
	*Aster tripolium*	leaves	halophyte	total	258 mM	1.4 weeks	≈1.3 ^#^	[41]
	*Aster tripolium*	leaves	halophyte	total	517 mM	1.4 weeks	≈1.5 ^#^	[41]
	*Sesuvium portulacastrum*	leaves	halophyte	total	428 mM	1.4 weeks	≈1.6 ^#^	[41]
	*Sesuvium portulacastrum*	leaves	halophyte	total	856 mM	1.4 weeks	≈2.1 ^#^	[41]
	*Thellungiella halophila*	leaves	halophyte	total	100 mM	5 days	1.5 *	[37]
	*Thellungiella halophila*	leaves	halophyte	total	200 mM	5 days	1.7 *	[37]
	*Thellungiella halophila*	leaves	halophyte	total	300 mM	5 days	1.8 *	[37]
MGDG/DGDG	*Arabidopsis thaliana*	leaves	salt-sensitive	total	100 mM	5 days	1.2 ^#^	[37]
	*Arabidopsis thaliana*	leaves	salt-sensitive	total	200 mM	5 days	1.7 ^#^	[37]
	*Sulla coronaria*	leaves	salt-sensitive	total	200 mM	2.9 weeks	≈0.9	[40]
	*Sulla carnosa*	leaves	salt-sensitive	total	200 mM	2.9 weeks	≈3.1 *	[40]
	*Thellungiella halophila*	leaves	halophyte	total	100 mM	5 days	1.3 ^#^	[37]
	*Thellungiella halophila*	leaves	halophyte	total	200 mM	5 days	1.4 ^#^	[37]
	*Thellungiella halophila*	leaves	halophyte	total	300 mM	5 days	1.4 ^#^	[37]

^a^ Abbreviations: GL: Glycolipids; MGDG: Monogalactosyldiacyloglycerol; DGDG: Digalactosyldiacyloglycerol; SQDG: Sulfoquinovosyldiacylglycerol; PM: Plasma membrane. ^b^ Salt-sensitive and salt-tolerant in this column refer to glycophytes’ salt tolerance level. ^c^ Average of three salt-sensitive cultivars is used. ^d^ Average of two salt-tolerant cultivars is used. ^e^ bundle sheath is relatively salt tolerant compare to mesophyll. *: Lipid content (LC) of salt-treated samples is significantly different from control. ^#^: Significance not specified. ≈: Estimated based on graphs.

**Table 4:** Glycerolipid composition (mol%) of plant (alga) membranes ^a^.

Species	Tissue	Salt Tolerant Level ^b^	Membrane Preparation Method	Membrane Class	PA	PS	PI	PC	PE	PG	MGDG	DGDG	SQDG	Ref.
*Spinacia oleracea*	leaves	salt-sensitive	Two-phase partitioning	PM	6.1	-	-	25.4	24.4	3.1	nd	2.7 ^c^	-	[64]
*Brassica oleracea*	buds	salt-sensitive	Two-phase partitioning	PM	13.1	-	-	17.6	13.7	3.4	0.3 ^c^	1.0 ^c^	-	[64]
*Hordeum vulgare*	leaves	salt-sensitive	Two-phase partitioning	PM	9.2	-	-	17.5	10.8	2.1	3.7 ^c^	1.4 ^c^	-	[64]
*Hordeum vulgare*	roots	salt-sensitive	Two-phase partitioning	PM	1.7	-	-	8.2	6.8	0.8	1.0 ^c^	0.3 ^c^	-	[64]
*Hordeum vulgare*	roots	salt-sensitive	density gradient centrifugation	PM	5.6	6.6	5.1 ^d^	12.7	33.8	3.6	-	-	-	[65]
*Hordeum vulgare*	roots	salt-sensitive	density gradient centrifugation	ER	1.0	1.5	9.8 ^d^	59.5	20.6	6.9	-	-	-	[65]
*Hordeum vulgare*	roots	salt-sensitive	density gradient centrifugation	Tonoplast + Golgi	1.8	2.5	7.4 ^d^	57.6	23.2	6.3	-	-	-	[65]
*Zea mays*	roots	salt-sensitive	Two-phase partitioning	PM	6.8	13.6	12.3	20.4	13.6	21.1	-	-	-	[31]
*Vigna radiata*	hypocotyl	salt-sensitive	density gradient centrifugation	PM	8.0	1.5	2.6	16	18.6	2.2	0.2 ^c^	0.8 ^c^	-	[66]
*Vigna radiata*	hypocotyl	salt-sensitive	density gradient centrifugation	Tonoplast	1.1	2.2	5.7	23.7	16.0	2.3	1.0 ^c^	3.4 ^c^	-	[66]
*Triticum aestivum*	roots	salt-sensitive	Two-phase partitioning	PM	13.1	9.2	5.9	18	10	28.3	-	-	-	[32]
*Zea mays*	roots	salt-tolerant	Two-phase partitioning	PM	7.5	19.4	14.9	22.4	19.4	16.4	-	-	-	[31]
*Dunalieila salina* (alga)	cell culture	halotolerant	density gradient centrifugation	PM	-	-	-	13.2	10.7	5.3	3.1 ^c^	2.9 ^c^	1.7 ^c^	[67]

^a^ Abbreviations: PC: Phosphatidylcholine; PE: Phosphatidylethanolamine; PS: Phosphatidylserine; PI: Phosphatidylinositol; PG: Phosphatidylglycerol; PA: Phosphatidic acid; MGDG: Monogalactosyldiacyloglycerol; DGDG: Digalactosyldiacyloglycerol; SQDG: Sulfoquinovosyldiacylglycerol; PM: Plasma membrane; ER: Endoplasmic reticulum. ^b^ Salt-sensitive and salt-tolerant in this column refer to glycophytes’ salt tolerance level. -: Not measured. nd: Not detected. ^c^ Glycolipids components (MGEG, DGDG, and SQDG) in plasma membrane and tonoplast might be contaminations from chloroplast membrane. ^d^ Values are the sum of PI and lysophosphatidylethanolamine.

**Table 5:** Fatty acid composition (mol%) of plant (alga) membranes.

Species	Tissue	Salt Tolerant Level ^a^	Membrane Type	14:0	16:0	16:1	17:0	18:0	18:1	18:2	18:3	20:0	20:1	20:2	20:3	22:0	22:1	Ref.
*Arabidopsis thaliana*	leaves	salt-sensitive	total	-	22.3	2.5	-	22.8	22.7	7.8	21.9	-	-	-	-	-	-	[37]
*Spinacia oleracea*	leaves	salt-sensitive	plasma membrane	-	29.7	1.4	-	3.2	10.4	27.1	27.3	nd	1.0	-	-	nd	-	[64]
*Brassica oleracea*	inflorescences	salt-sensitive	plasma membrane	-	20.8	1.6	-	2.4	13.4	17.4	42.3	1.4	0.7	-	-	tr	-	[64]
*Hordeum vulgare*	leaves	salt-sensitive	plasma membrane	-	31.5	0.9	-	4.1	3.1	32.9	25.8	tr	tr	-	-	1.8	-	[64]
*Hordeum vulgare*	roots	salt-sensitive	plasma membrane	-	43.9	1.5	-	2.7	2.3	33.5	12.8	0.4	2.1	-	-	0.9	-	[64]
*Zea mays*	roots	salt-sensitive	plasma membrane	-	19.2	-	5.5	21.7	27.6	7.1	-	18.5	-	-	-	-	-	[31]
*Vigna radiata*	hypocotyl	salt-sensitive	plasma membrane	-	35.0	-	-	5.9	9.2	21.4	19.0	-	1.6	1.6	2.2	-	1.1	[66]
*Vigna radiata*	hypocotyl	salt-sensitive	Tonoplast	-	39.4	-	-	6.1	9.1	22.2	19.8	-	1.5	1.2	0.8	-	2.1	[66]
*Avena sativa*	roots	salt-sensitive	plasma membrane	nd	22.5	tr	-	tr	3.0	48.0	25.0	-	-	-	-	-	-	[68]
*Triticum aestivum*	roots	salt-sensitive	plasma membrane	-	17.4	9.6	13.2	13.6	12.3	14.6	-	19.3	-	-	-	-	-	[32]
*Zea mays*	roots	salt-tolerant	plasma membrane	-	6.1	-	26.3	4.1	23.4	14.6	-	25.3	-	-	-	-	-	[31]
*Boea hygroscopica*	leaves	not sure	thylakoid membranes	2.0	28.0	2.0		7.0	8.0	36.0	17.0	-	-	-	-	-	-	[69]
*Thellungiella halophila*	leaves	halophyte	total	-	41.0	6.0	-	23	7.7	0.9	21.3	-	-	-	-	-	-	[37]
*Spartina patens*	Callus tissue	halophyte	plasma membrane	-	12.9	-	0.3	0.8	-	15.8	-	0.5	-	-	-	1.0	-	[36]

^a^ Salt-sensitive and salt-tolerant in this column refer to glycophytes’ salt tolerance level. -: Not measured. nd: Not detected. Tr: Trace.

**Table 6:** Lipid metabolism proteins regulated by salt stress.

Protein	Organism	Protein Abundance	Transcript Regulation	Ref.
acyl carrier proteins 1	*Thellungiella halophila; Arabidopsis thaliana*		down	[165]
acyl carrier proteins 4	*Thellungiella halophila; Arabidopsis thaliana*		down	[165]
acetyl-CoA carboxylase carboxyl transferase subunit beta	*Oleaginous Diatom*		up	[173]
carboxyltransferase β -subunit	*Chlamydomonas* sp. JSC4		up	[174]
dihydrolipoyllysine-residue acetyltransferase 2	*Thellungiella halophila*; *Arabidopsis thaliana*		down	[165]
biotin carboxylase	*Chlamydomonas reinhardtii*	decreased		[175]
	*Cucumis sativus*	increased		[167]
	*Medicago truncatula*	decreased		[166]
	*Thellungiella halophila*		up	[165]
	*Chlamydomonas* sp. JSC4		up	[174]
long-chain acyl-CoA synthetase 2	*Thellungiella halophila*		up	[165]
ketoacyl-ACP reductase	*Synechocystis* sp. PCC		up	[176]
enoyl-ACP reductase	*Thellungiella halophila*; *Arabidopsis thaliana*		down	[165]
	*Oryza sativa*	increased		[169]
glyceraldehyde-3-phosphate dehydrogenase	*Oryza sativa*		up	[171]
	*Pleurotus sajor-caju*		up	[177]
glycerol-3-phosphate dehydrogenase	*Dunaliella salina*		down	[170]
	*Suaeda salsa*		up	[178]
glycerol-3-phosphate acyltransferase	*Lepidium latifolium*		down	[179]
UDP-glucose pyrophosphorylase	*Solanum lycopersicum*		up	[180]
	*Hordeum vulgare*		up	[181]
	*Oryza sativa*		down	[182]
phosphoethanolamine *N*-methyltransferase	*Zea mays*		up	[183]
	*Sugar beet monosomic addition line*	increased		[184]
choline kinase	*Spinacia oleracea*	no-change		[185]
non-specific phospholipase C4	*Arabidopsis thaliana*		up	[186]
non-specific phospholipase C5	*Arabidopsis thaliana*		up	[187]

## Appendix A

**Table A1:** Genetic engineering of fatty acid desaturases and the effect on plant salt tolerance.

Gene	Organism	Mutant/Overexpression	Salt Tolerance (Compared to WT)	Cellular Changes (Compared to WT)	Ref.
*FAD2*	*Arabidopsis thaliana*	*fad2* mutant	more sensitive during seed gemination and early seedling growth	lower polyunsaturation reduced Na+/H+ exchange activity higher Na+ accumulation in the cytoplasm of root cells	[206]
*FAD3*	*Lycopersicon esculentum*	sense overexpression	enhanced tolerance	increased 18:3 FA decreased 18:2 FA improved maintenance of membrane integrity higher SOD and APX activity in the chloroplast	[207]
		antisense overexpression	reduced tolerance	increased 18:2 FA decreased 18:3 FA	[207]
*FAD6*	*Arabidopsis thaliana*	*fad6* mutant	reduced tolerance	greater Na+ accumulation lower K+ accumulation increased electrolyte leakage rate and malondialdehyde production (more severe oxidative damage) decreased activities of anti-oxidative enzymes	[208]

SOD: superoxide dismutase. APX: ascorbate peroxidase.
